# What barriers do people experience to engaging in the arts? Structural equation modelling of the relationship between individual characteristics and capabilities, opportunities, and motivations to engage

**DOI:** 10.1371/journal.pone.0230487

**Published:** 2020-03-25

**Authors:** Daisy Fancourt, Hei Wan Mak

**Affiliations:** Department of Behavioural Science and Health, University College London, London, United Kingdom; University of Birmingham, UNITED KINGDOM

## Abstract

**Objectives:**

Participation in the arts has well-documented benefits for health. However, participation in the arts is socially patterned, and it remains unclear why this is: what factors act as barriers or enablers of individual arts engagement. Therefore this study explored how individual characteristics predict individuals’ capabilities, opportunities and motivations to engage in participatory arts activities.

**Methods:**

We analysed data from 6,867 adults in the UK (61.2% female, average age 46.7 years) who engage infrequently in performing arts, visual arts, design and crafts, literature-related activities, or online, digital and electronic arts. We constructed a structural equation model to explore the relationship between demographic factors (including age, sex, ethnicity or socio-economic status), health factors (including physical and mental health) or social factors (including living alone, urban density, loneliness or socialising) and perceived barriers to arts engagement.

**Results:**

Individuals with poorer physical and mental health experienced more barriers affecting their perceived capabilities to engage in the arts, whilst individuals with poorer mental health also described experiencing more barriers affecting their motivations to engage. Individuals of lower SES reported more barriers in terms of opportunities to engage, whilst loneliness was related to more barriers around opportunities and motivations and living alone was associated with more opportunity barriers. Interestingly, adults who were older experienced fewer barriers relating to capabilities or opportunities, as did men, whilst being of white ethnicity was associated with fewer barriers across all three domains. Adults who were more socially engaged or who had poorer physical health experienced fewer barriers relating to motivations. Geographical area of dwelling was not related to any barriers.

**Conclusions:**

This study has shown for the first time where the barriers leading to differential patterns of arts engagement lie. The findings could inform future behaviour change interventions designed to encourage arts engagement amongst individuals who are least likely to engage.

## Introduction

Participation in the arts has wide-ranging benefits for the prevention and management of mental and physical health conditions as well as supporting broader determinants of health [1]. However, participation in the arts is socially patterned. Recent analyses of predictors of arts engagement in the UK have highlighted that there is a strong social gradient across arts participation, with those with fewer educational qualification, from families of lower socio-economic status (SES) and with lower household income less likely to engage [2]. This echoes the findings from some reports, which have highlighted socio-economic factors as barriers to participation [3–5]. There is also some evidence that demographic factors such as age, sex and ethnicity affect participation rates, but the evidence here is more nuanced. For example, participation has been found to be lower amongst older adults, especially for those over 85 [2,6], and higher amongst women, especially for engaging in performing arts activities [2]. Regarding ethnicity, individuals of certain ethnic minority groups such as people who are Asian/Asian British are less likely to engage the arts, but people who are of Black/Black British ethnicity are more likely to engage in certain activities such as performing arts activities [2]. Thus it is clear that participation is affected by a range of individual factors.

However, what remains unclear is why these individual factors act as barriers or enablers of individual arts engagement. Human behaviour can be understood through applying theories and models of behaviour change. Whilst specific theories of behaviour change vary across disciplines [7], there have been efforts in recent years to identify a minimum set of constructs can be taken to represent key influences on behaviour. Specifically, COM-B is an integrated theoretical model that proposes that three sets of factors influence individuals’ behaviour: capability to engage (i.e. knowledge and skills), opportunity to engage (in an individual’s social and physical environment), and motivation to engage (both reflective and automatic) [8]. Applying this to arts participation could help us to understand why differences in individual participation exist.

For example, lower patterns of engagement amongst individuals of lower SES could be due to lower physical capability (e.g. not having an artistic skill such as knowing how to play a musical instrument), psychological capability (e.g. not knowing enough about activities one could engage in) or physical opportunity (e.g. not living in an area where there are arts activities to engage with or not having enough money to pay for arts classes or transport to arts venues). As socio-economic burden is experienced disproportionately more by individuals with poor health, it is possible that individuals with poor mental or physical health may face more socio-economic difficulties or live in areas with fewer activities available, thereby facing more barriers relating to opportunities [9]. But it is also possible that barriers relating to health are in fact to do with differences in capabilities or motivations. Indeed, previous research has shown that conditions such as anxiety can affect concentration [10], whilst poor mental health is associated with low self-esteem [11], both of which would affect psychological capabilities to engage in the arts. Further, physical illness can be associated with fatigue (another component of psychological capability), and disability can be associated with experiencing physical barriers to accessing the arts [12,13], which would affect physical capabilities to engage. Common features of poor health such as social anxiety and behavioural futility are also both well-known barriers to engagement in any kinds of productive activities [14,15], so could lead to motivational barriers. When considering social factors, loneliness and isolation may lead to barriers to engaging in the arts. Studies have found a relationship between arts engagement of peers and spouses and an individual’s own level of engagement, suggesting social factors can influence social opportunity to engaging in arts activities [16]. Further, loneliness is associated with lower perceived control, autonomy and attribution, which may affect motivations to engage [17,18].

However, whilst these demographic, health-related and social factors may by theorised to be related to barriers to arts engagement, whether such a relationship exists remains untested in practice. Understanding predictors of barriers to engagement is crucial to being able to develop interventions to address and remove differences in participation. Consequently, this study applied the lens of COM-B to explore whether demographic factors (including age, sex, ethnicity or socio-economic status), health factors (including physical and mental health) or social factors (including living alone, urban density, loneliness or socialising) predict individuals’ capabilities, opportunities and motivations to engage in participatory arts activities. Specifically the study focused on a sample of adults in the UK who engaged infrequently in creative activities. As this study focused on a number of interrelated factors, we used a structural equation modelling approach involving a large sample of adults that allows us to simultaneously model the relationships between all included variables.

## Materials and methods

### Procedure

We used data from the *Feel Good* data set: a sample of 43,084 adults (aged 18 and above) living in the UK. The data were gathered from May to June 2019 as part of a Citizen Science experiment run by the British Broadcasting Corporation (BBC). The study was promoted through the BBC Arts website as part of the UK’s annual ‘Get Creative Festival’ and individuals participated by completing an online survey that lasted approximately 20 minutes. For these analyses, we excluded individuals who had taken the test previously (n = 265), and individuals who had provided incomplete data (n = 11,182). As this study explored barriers to engagement, we focused on individuals who had low levels of engagement that could be indicative of experiencing barriers (whether psychological, social or physical). We therefore restricted our sample to individuals who were “infrequently” engaged (taking part in activities either on their own or with others less than once a month). This left a sample size of 6,867.

Participants were 61.2% female, with an average age of 46.7 years (SD = 13, range 18–90), majority white British or Irish (86.8%). Participants provided data on a wide range of variables including demographic, socio-economic, health and social factors. The original study was approved by UCL Research Ethics Committee (reference 14895/003) and all participants gave informed consent to data collection and use of the data in subsequent analyses.

### Measures

**Participatory arts activities** were defined in the dataset following a theorised model for population-level research as participatory activities consisting of performing arts, visual arts, design and crafts, literature-related activities, and online, digital and electronic arts [19]. Participants were asked how often they took part in any of the following activities: singing (either at home or in a choir), dancing (such as ballroom dancing/salsa classes), playing a musical instrument (either practising at home or in a band or orchestra), rehearsing or performing in a play/drama/opera/musical theatre, painting, drawing, printmaking, sculpture on your own, photography, pottery, calligraphy or jewellery making, textile crafts such as embroidery, crocheting or knitting, wood crafts such as carving or furniture making, reading a novel, stories, poetry or plays for pleasure (either alone or in a book club), creative writing, creating artworks or animations on a computer, and making films or videos. Further, in line with some previous evidence syntheses [20], we extended this definition to include gardening and baking or cooking as they are also creative activities that could be considered artistic. Although individuals’ decisions on whether or not to engage in any one specific arts activity are driven by a range of factors including perceived feelings of resonance, meaning and identity from an activity [21], engagement with the arts in general is considered to be an innate human behaviour [22]. So to allow flexibility for individual preference, we explored ‘arts activities’ as a collective.

**Barriers to engagement** were measured using an 18-item scale developed based on the COM-B Self-Evaluation Questionnaire [23]. Individuals were asked to select in binary form barriers that would need to be overcome for them to engage more frequently in arts activities, with three questions each to represent psychological capabilities, physical capabilities, social opportunities, physical opportunities, automatic motivations and reflective motivations. For example, to measure barriers relating to physical opportunity participants answered yes/no to the item: “In order to engage more in arts activities, I would need to have more time to do it (e.g. having time to yourself or capacity away from other commitments).” Participants were given a point for each barrier they identified as relevant to them, so as each of the six categories had 3 questions, this provided a score from 0–3 for each category. As there were two categories in each of the domains of capabilities, opportunities and motivations, this provided an overall score of 0–6 for motivations each of these three domains, with a higher score indicating the presence of more barriers. The total 18-item scale had a Cronbach’s alpha of 0.85, with 6-item subscale alphas of 0.63 for capabilities, 0.66 for opportunities, and 0.73 for motivations. The full scale is available in the supplementary material.

**Individual demographic factors** included age (categorised as 18–30, 31–49, 50–64, and 65+), sex and ethnicity (white British vs other). Socio-economic status was assessed using three variables: educational attainment (no formal qualifications, qualifications to age 16/GCSEs/O-levels, qualifications to age 18/A-levels, degree or post-school qualifications, or postgraduate degree), household income (<£16,000, £16,000-£29,999, £30,000-£59,999, £60,000-£89,999, £90,000-£119,999, or >£120,000), and employment status (in full-time employment, in part-time employment, retired or not working). Physical health was assessed using three variables: presence of any chronic or long-standing illness (self-reported yes or no), presence of chronic pain (self-reported as none, mild, moderate or severe pain), or presence of any problems affecting mobility (self-reported yes or no). Mental health was also assessed using three variables: depression (measured as a continuous variable using the 8-item Centre for Epidemiologic Studies Depression Scale (CES-D) with scores from 0–8 and higher scores indicating more depressive symptoms [24]), anxiety (measured as a continuous variable using the 7-item Generalised Anxiety Disorder Questionnaire (GAD-7) with scores from 0–21 and higher scores indicating greater anxiety [25]), and stress (measured using the single-item question ‘on average, how stressed would you say you feel’ with responses from 0–10; higher scores indicating higher stress). We further measured the type of area of dwelling (self-reported city, town, village or isolated dwelling), frequency of socialising with friends or family (once or twice a year, every few months, once or twice a month, once or twice a week, three or more times a week), loneliness (using the UCLA 3-item Loneliness Scale with scores from 3–9 and higher scores indicating higher loneliness [26]), and whether individuals lived alone or with others.

### Construction of the structural equation models

**Fig 1:**
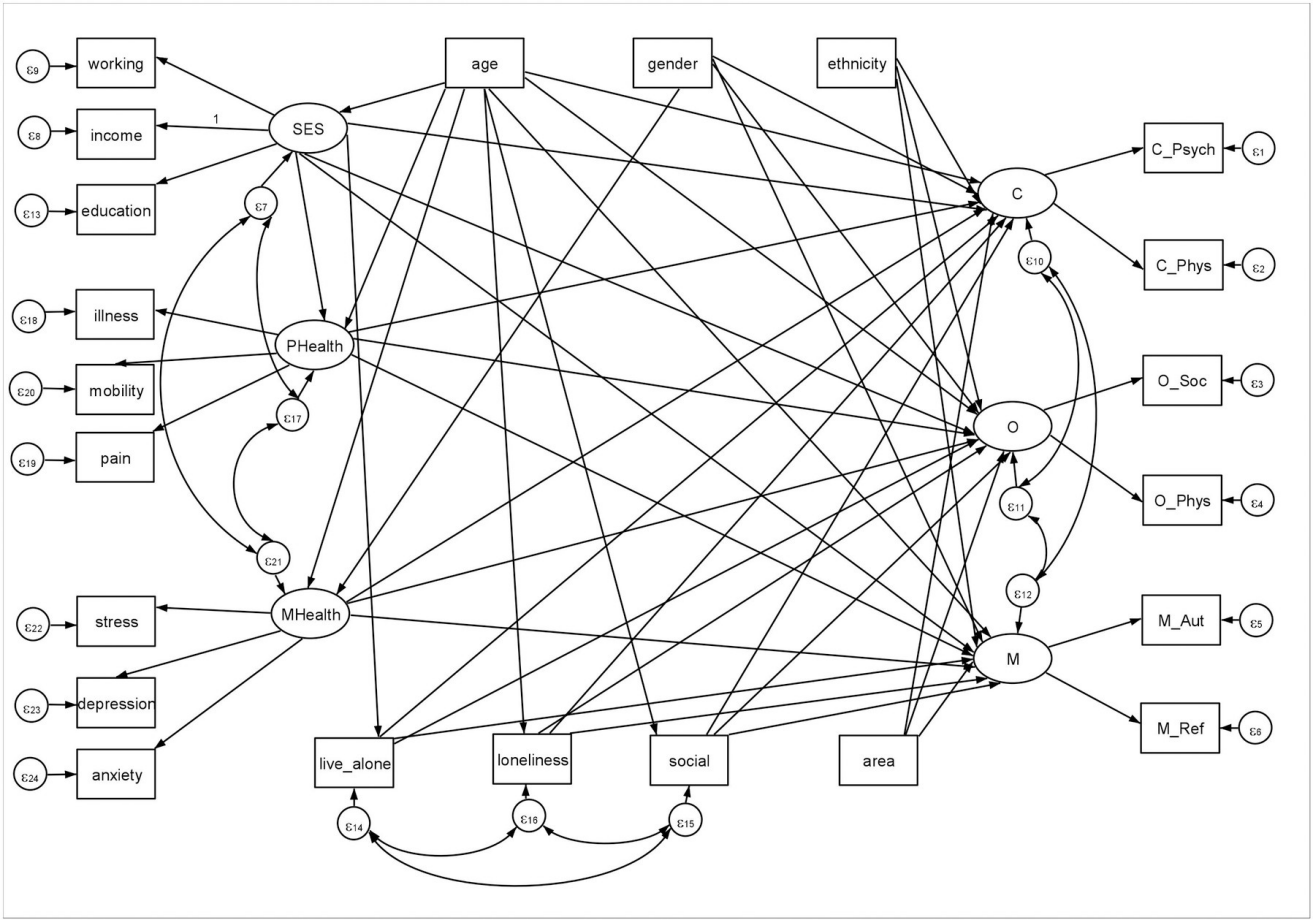
Hypothetical model linking demographic factors, health factors and social factors with individuals’ capabilities, opportunities and motivators to engage in participatory arts activities. C = capabilities, O = opportunities, M = motivations, SES = socio-economic status, P Health = physical health, M Health-mental health, C_Psych = psychological capabilities, C_Phys = physical capabilities, O_Soc = social opportunities, O_Phys = physical opportunities, M_Aut = automatic motivations, M_Ref = reflective motivations, ε = error.

Determining the direction of an association in SEM can be challenging [27]. Age, sex and ethnicity are inevitably exogenous so can only act as influencers of other factors but cannot be influenced themselves. Were we exploring predictors of arts engagement itself, we might assume bidirectional relationships between arts engagement and the other socio-economic, health, and social variables in our model. However, as this SEM in fact explored predictors of perceived capabilities, opportunities and motivations (i.e. barriers of arts engagement rather than arts engagement itself), we assumed that these barriers were the result of socio-economic health and social factors. It is possible that certain barriers (such as not have activities close by) might contribute to an individual’s feelings of loneliness, so there may be some bidirectional relationship. But in large part the direction of the relationship is likely to be from tangible components of socio-economic status, mental and physical health, and social behaviours to the resulting perception of barriers to engaging in the arts, so we specifically focused on the relationship as uni-directional (Fig 1). It is possible that further interconnections between our demographic and engagement factors could exist, but in order to avoid overloading the model, we focused in particular on the paths going to capabilities, opportunities and motivations. We further provide the correlation matrix (S1 Table) for readers to consider how the model could be reworked using different assumptions.

### Statistical analysis

Analyses were carried out in Stata (Version 14, StataCorp). We fitted an SEM to determine the relationship between demographic, socio-economic and social variables and barriers to engagement. We used maximum likelihood estimation and, as there was some violation of multivariate normality, we applied the Satorra Bentler estimator to obtan Satorra Bentler standard errors. We ran the model using all hypothesised paths. There was no evidence of multicollinearity (as assessed using variance inflation factors) and no outliers. 143 cases were excluded due to missing data. We report the Chi-square test results from Satorra-Bentler scaled statistics. However, in ascertaining the model fit, as the chi-square test is very sensitive to sample size (and therefore when the sample size is large very small differences between the observed and reproduced covariance matrices will result in a statistically significant chi-square value), we used a number of factors [28]. This included the Root Mean Square Error of Approximation, and Standardised Root Mean Square Residual, the Comparative Fit Index, and the coefficient of determination (CD) [29].

For all factors included in the SEM, higher scores indicate older age, female sex, white ethnicity, being of higher SES, having more physical health problems, having more mental health problems, living alone, being lonely, socialising frequently, living in a more isolated location, and experiencing more barriers relating to capabilities, opportunities or motivations. Given there is no consensus on what constitutes a large, medium or small association in SEM, for this study we considered that *β* values of ≥0.2 had the greatest importance and they are shown as thick black lines, *β* values of ≥0.1 were taken as being of moderate importance and are shown as medium black lines, and smaller significant *β* values were taken as being of lesser importance and are shown as thin black lines. Non-significant paths are not shown in the SEM figure.

## Results

**Fig 2:**
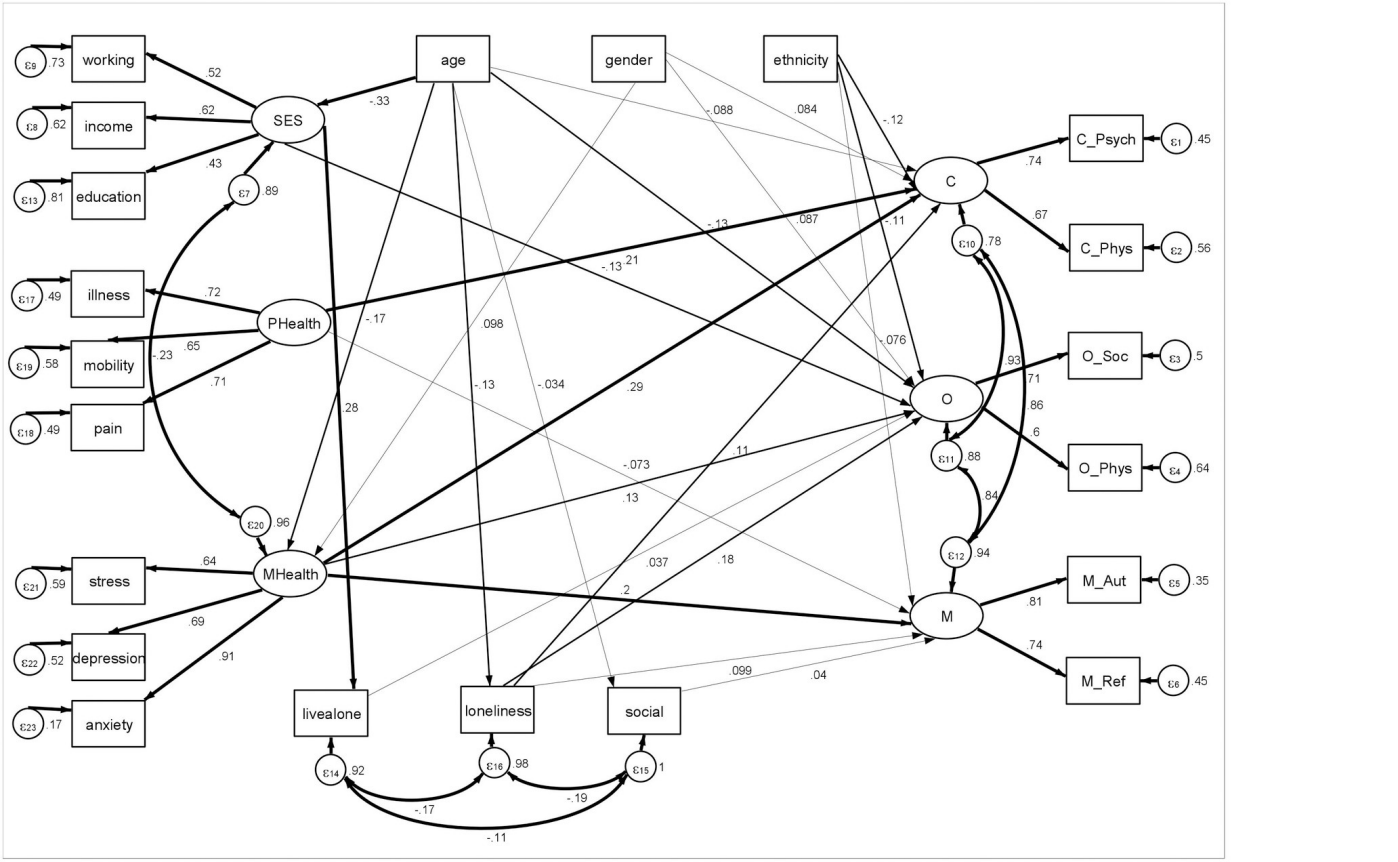
Structural equation model of demographic factors, health factors and social factors in relation to individuals’ capabilities, opportunities and motivations to engage in participatory arts activities. C = capabilities, O = opportunities, M = motivations, SES = socio-economic status, P Health = physical health, M Health-mental health, C_Psych = psychological capabilities, C_Phys = physical capabilities, O_Soc = social opportunities, O_Phys = physical opportunities, M_Aut = automatic motivations, M_Ref = reflective motivations, ε = error.

Participants showed a good spread across all demographic, health-related and social factors (Table 1). Fig 2 shows the full SEM. Full coefficients, p values and standardised Betas are shown in S2 Table. The resulting model was an acceptable fit for the data (χ^2^ = 6,407.82, df = 163, *p <* .001, RMSEA = 0.075, SRMR = 0.070, CFI = 0.82, CD = 0.29).

**Table 1:** Participant demographics.

Demographics	N = 6,825	Social	
Sex, female	61.2%	Live alone	17.2%
Age, μ (SD)	46.7 (13.0)	Lonely	
Ethnicity, White British/Irish/Other %	86.8%	Hardly ever/never	32.4%
Geographical area of dwelling		Some of the time	47.4%
City	32.9%	Often	20.2%
Town	42.8%	Frequency of socialising	
Village	20.3%	Once or twice a year	9.9%
Isolated	4.1%	Every few months	21.4%
Educational attainment		Once or twice a month	33.3%
No qualifications	4.2%	Once or twice a week	28.8%
GCSE/CSE/O-levels or other age 16 attainment	12.4%	Three or more times a week	6.7%
A-levels or other post-16 attainment	14.7%	**Number of barriers to engagement reported**	
Undergraduate degree	43.0%	Psychological capabilities	
Postgraduate degree	25.7%	0	13.1%
Occupational status		1	21.7%
In employment/study	79.8%	2	32.2%
Retired/not working	20.2%	3	32.9%
Household income		Physical capabilities	
<£16,000	11.0%	0	9.0%
£16,000-£29,999	20.5%	1	29.6%
£30,000-£59,000	35.3%	2	43.9%
£60,000-£89,000	18.4%	3	17.5%
£90,000-£119,999	7.8%	Social opportunities	
>£120,000	7.0%	0	24.4%
**Health**		1	23.2%
Chronic physical illness/disability/infirmity	18.0%	2	25.0%
Chronic pain		3	27.4%
None	60.6%	Physical opportunities	
Mild	26.5%	0	10.8%
Moderate	10.4%	1	23.2%
Severe	2.6%	2	29.8%
Mobility issues	6.3%	3	36.2%
Anxiety, μ (SD)	6.8 (5.3)	Automatic motivations	
Depression, μ (SD)	3.8 (2.7)	0	6.7%
Stress, μ (SD)	7.0 (2.3)	1	17.1%
		2	27.3%
		3	48.9%
		Reflective motivations	
		0	10.8%
		1	19.5%
		2	29.2%
		3	40.6%

### Demographic factors

The SEM showed that there were only very small associations between age, sex and ethnicity and the number of barriers relating to capabilities, opportunities and motivations experienced by individuals. SES showed a modest association with opportunities, with individuals of higher SES experiencing fewer social and physical opportunity barriers (*β* = -0.13, p < .001).

### Health factors

Physical health showed one of the strongest associations with barriers relating to capabilities (*β* = 0.21, p < .001), with individuals with more physical health problems experiencing more capability barriers. However, physical health also had a weak association with barriers relating to motivations (*β* = -0.073, p = .001), with individuals with more physical health problems in fact experiencing fewer motivation barriers. Physical health was not associated with opportunity barriers. For mental health, there was a strong association with both barriers relating to capabilities (*β* = 0.29, p < .001) and motivations (*β* = 0.20, p < .001), and a modest association with barriers relating to opportunities (*β* = 0.13, p < .001), with individuals with more mental health problems experiencing more barriers.

### Social factors

There were modest associations between loneliness and barriers to engagement. Being lonely was associated with more barriers relating to capabilities (*β* = 0.11, p < .001), opportunities (*β* = 0.18, p < .001), and motivations (*β* = 0.099, p < .001). There was only a weak association between living alone and barriers relating to opportunities (*β* = 0.037, p = .02) and no association with barriers relating to capabilities or motivations. More frequent social contact was weakly associated with more motivational barriers (*β* = 0.04, p = .006), but not with capabilities or opportunities. Whether an individual lived in a rural or urban area was not associated with any barriers.

## Discussion

Overall, this study showed that the clearest predictors of barriers to engaging in the arts related to health. Individuals with poorer physical and mental health experienced more barriers affecting their perceived capabilities to engage in the arts, whilst individuals with poorer mental health also described experiencing more barriers affecting their motivations to engage. Amongst smaller associations, individuals of lower SES reported more barriers in terms of opportunities to engage, whilst loneliness was related to more barriers around opportunities and motivations and living alone was associated with more opportunity barriers. Interestingly, adults who were older experienced fewer barriers relating to capabilities or opportunities, as did men, whilst being of white ethnicity was associated with fewer barriers across all three domains. Additionally, adults who were more socially engaged or who had poorer physical health experienced fewer barriers relating to motivations. Geographical area of dwelling was not related to any barriers.

The main finding from this study was that health appeared to act as a clear source of barriers to engaging in the arts. This echoes findings from previous papers that have shown lower participation in the arts amongst individuals with illness or disability, independent of factors such as socio-economic status [3,30]. However, it expands on these findings by showing specifically where the barriers lie. It is notable that capabilities are specifically affected by both mental and physical health, as theorised. It appears important to address these barriers, as other research has shown that individuals with mental illness (specifically depression) can experience the same benefits for emotion regulation from arts engagement as individuals without depression, even if emotional responses to other activities are affected by their mental health [31]. However, it is also notable that, although individuals with poorer mental health were less motivated to engage in the arts, individuals with poorer physical health were actually more motivated to engage. This suggests either that individuals are aware of benefits from arts engagement either for their health, sense of self or social benefits, or that the arts provide enjoyment. Indeed, previous studies have discussed the benefits of the flexibility of different modes of engagement as an enabling factor for engagement [32,33]. Finally, we found that mental illness was related to experiencing more barriers relating to opportunities to engage, although this relationship was weaker than for other types of barriers. However, another explanation is that an individual with poor mental health may have just as many physical and social opportunities to engage, but simply perceived that there are more barriers as manifestations of their mental health conditions. As such, future research is needed to identify whether interventions providing more opportunities for individuals with poor mental health, or interventions that reframe existing opportunities to better encourage participation are most needed.

It is also interesting that SES had a weaker relationship with barriers to engagement than health. Whilst there is a recognised social gradient across arts engagement, as explored theoretically and demonstrated empirically [2,34], it is of note that this study focused on participating in the arts rather than attending cultural venues, and specifically focused on home-based as well as community-based activities, using a broad and inclusive definition incorporating varied art forms and modes of participation. As such, it is possible that cultural attendance, which requires proximity to venues, may be associated with more barriers. This same point could explain our finding that geographical area was not related to barriers: whilst geographical area (including level of urbanisation) is a predictor of cultural engagement, it has been shown not to be an independent predictor of arts participation (paper under review).

In relation to social factors, the fact that loneliness and living alone were related to more opportunity barriers is as theorised. However, the converse finding that frequent social interaction is associated with higher motivation suggests that those individuals who are already socially active are motivated to engage in other activities may have already overcome barriers to engaging in activities generally. Finally, our finding that those who are female and of white ethnicity experience fewer barriers to engagement supports a number of previous studies that have shown higher engagement amongst these groups [2,16]. However, it expands on previous work by showing it is capabilities, opportunities and motivations that are all affected by sex and ethnicity. This suggests a multifaceted approach is required to increase engagement from men and ethnic minority groups. Our finding that there are in fact fewer barriers as people age goes against some previous research [2,6]. However, our study focused on age as a continuous variable, suggesting that although age does not affect motivation to engage, work and family pressures in younger adulthood may limit opportunities and capabilities. Whether more barriers emerge in older age specifically remains to be explored further.

This study has a number of strengths, including its use of a large sample, its inclusion of a rich set of variables on barriers to arts engagement and theory-driven approach to behaviours, and its broad range of variables included within the SEM. However, as the data are cross-sectional, causality cannot be determined. Although the number of participants is large and they showed socio-economic and demographic diversity, the sample is not nationally representative. Further, we used self-report for all variables, so responses may include individual bias. Finally, we looked at perceived barriers to arts engagement at a single moment in time. Whether and how perceived barriers are affected by life events remains to be explored further. Future studies may also like to expand the focus from arts participation to also include engagement with culture or heritage. Further, this study focused on behavioural *intentions*. This suggests that if certain factors could be addressed, people would engage more in arts activities. However, whether addressing these barriers does lead to increased engagement in practice remains to be examined in future studies.

In conclusion, this study built on previous studies showing differences in arts engagement based on demographic, health-related and social factors by elucidating where the barriers leading to these differential patterns of engagement lie. In particular, mental and physical health are related to capabilities and motivations to engaging in home- and community-based arts activities, while SES and social factors are related to further opportunity and motivational barriers. The identification of these barriers could inform future behaviour change interventions designed to encourage engagement with arts activities amongst individuals who are currently less likely to engage. Given, in particular, previous research showing barriers to engagement amongst those with mental or physical illness and the strong evidence base showing the benefits of engagement for health, the findings presented here could inform current social prescribing schemes underway internationally that are referring individuals to arts activities by highlighting specific barriers that need to be addressed to enable this engagement.

## Supporting information

S1 Table Correlation matrix of continuous or ordinal variables included in the SEM. All values show Pearson’s correlation coefficient (r) for linear variables and Spearman’s correlation (ρ) coefficient for ordinal variables. Boldface indicates significance p < .05.
(DOCX)

S2 Table Unstandardised B and standardised β coefficients and p values for the structural equation model. var = variance, cov = covariance.
(DOCX)
