# Supplementary material for: What barriers do people experience to engaging in the arts? Structural equation modelling of the relationship between individual characteristics and capabilities, opportunities, and motivations to engage
Source: PLoS One. 2020 Mar 25;15(3):e0230487. doi: 10.1371/journal.pone.0230487 (PMC7094843; doi:10.1371/journal.pone.0230487)
Supplement: S1 Table — All values show Pearson’s correlation coefficient (r) for linear variables and Spearman’s correlation (ρ) coefficient for ordinal variables. Boldface indicates significance p < .05. (DOCX) [file pone.0230487.s001.docx]

Supplementary Table 1: Correlation matrix of continuous or ordinal variables included in the SEM

|  | age | area | income | educ | pain | anx | depres | stress | lonel | social | CPsych | CPhys | OSoc | OPhys | MAut |
| --- | --- | --- | --- | --- | --- | --- | --- | --- | --- | --- | --- | --- | --- | --- | --- |
| age | 1.00 |  |  |  |  |  |  |  |  |  |  |  |  |  |  |
| area | **0.22** | 1.00 |  |  |  |  |  |  |  |  |  |  |  |  |  |
| income | **-0.09** | **-0.01** | 1.00 |  |  |  |  |  |  |  |  |  |  |  |  |
| education | **-0.19** | **-0.11** | **0.28** | 1.00 |  |  |  |  |  |  |  |  |  |  |  |
| pain | **0.22** | **0.07** | **-0.18** | **-0.14** | 1.00 |  |  |  |  |  |  |  |  |  |  |
| anxiety | **-0.15** | **-0.03** | **-0.14** | **-0.03** | **0.17** | 1.00 |  |  |  |  |  |  |  |  |  |
| depression | **-0.07** | **-0.03** | **-0.21** | **-0.06** | **0.20** | **0.63** | 1.00 |  |  |  |  |  |  |  |  |
| stress | **-0.17** | **-0.03** | -0.01 | **0.03** | **0.09** | **0.59** | **0.43** | 1.00 |  |  |  |  |  |  |  |
| loneliness | **-0.15** | **-0.06** | **-0.18** | -0.01 | **0.09** | **0.45** | **0.55** | **0.31** | 1.00 |  |  |  |  |  |  |
| socialising | **-0.05** | **-0.06** | **0.03** | **0.03** | **-0.10** | **-0.15** | **-0.19** | **-0.12** | **-0.17** | 1.00 |  |  |  |  |  |
| C_Psych | **-0.10** | **-0.06** | **-0.12** | **-0.05** | **0.06** | **0.28** | **0.27** | **0.18** | **0.23** | **-0.03** | 1.00 |  |  |  |  |
| C_Phys | **-0.04** | **-**0.02 | **-0.12** | **-0.08** | **0.27** | **0.27** | **0.27** | **0.17** | **0.18** | **-0.07** | **0.50** | 1.00 |  |  |  |
| O_Soc | **-0.05** | **-0.04** | **-0.10** | **-0.05** | **0.05** | **0.18** | **0.18** | **0.08** | **0.23** | **-0.04** | **0.50** | **0.37** | 1.00 |  |  |
| O_Phys | **-0.16** | **-0.04** | **-0.14** | -0.02 | **0.06** | **0.17** | **0.14** | **0.15** | **0.13** | **-0.05** | **0.43** | **0.38** | **0.43** | 1.00 |  |
| M_Aut | **-0.06** | **-0.04** | **-0.05** | **-0.03** | 0.02 | **0.18** | **0.18** | **0.10** | **0.15** | -0.01 | **0.51** | **0.39** | **0.49** | **0.36** | 1.00 |
| M_Ref | **-0.07** | **-0.04** | **-0.05** | **-0.07** | 0.01 | **0.18** | **0.16** | **0.09** | **0.15** | -0.01 | **0.46** | **0.37** | **0.48** | **0.34** | **0.59** |

All values show Pearson’s correlation coefficient (r) for linear variables and Spearman’s correlation (ρ) coefficient for ordinal variables. Boldface indicates significance p<.05
