# Supplementary material for: What barriers do people experience to engaging in the arts? Structural equation modelling of the relationship between individual characteristics and capabilities, opportunities, and motivations to engage
Source: PLoS One. 2020 Mar 25;15(3):e0230487. doi: 10.1371/journal.pone.0230487 (PMC7094843; doi:10.1371/journal.pone.0230487)
Supplement: S2 Table — var = variance, cov = covariance. (DOCX) [file pone.0230487.s002.docx]

Supplementary Table 2: Unstandardised B and standardised β coefficients and p values for the structural equation model

|  | B | β | P(>\|z\|) |
| --- | --- | --- | --- |
| Regressions: |  |  |  |
| Socialising |  |  |  |
| Age | -0.003 | -0.034 | 0.004 |
| _cons | 4.139 | 3.836 | <.001 |
| Loneliness |  |  |  |
| Age | -0.007 | -0.128 | <.001 |
| _cons | 2.207 | 3.095 | <.001 |
| Living alone |  |  |  |
| SES | 1.280 | 0.276 | <.001 |
| _cons | 7.791 | 2.050 | <.001 |
| SES |  |  |  |
| Age | -0.021 | -0.326 | <.001 |
| Capabilities (C) |  |  |  |
| Socialising | 0.012 | 0.017 | 0.237 |
| Loneliness | 0.113 | 0.108 | <.001 |
| Living alone | 0.002 | 0.010 | 0.504 |
| SES | -0.037 | -0.040 | 0.097 |
| Physical health | 0.570 | 0.209 | <.001 |
| Mental health | 0.150 | 0.293 | <.001 |
| Age | -0.005 | -0.088 | <.001 |
| Sex | 0.128 | 0.084 | <.001 |
| Ethnicity | -0.265 | -0.120 | <.001 |
| Geographical area | -0.016 | -0.018 | 0.201 |
| Opportunities (O) |  |  |  |
| Socialising | -0.005 | -0.007 | 0.633 |
| Loneliness | 0.202 | 0.181 | <.001 |
| Living alone | 0.008 | 0.037 | 0.020 |
| SES | -0.130 | -0.134 | <.001 |
| Physical health | 0.038 | 0.013 | 0.559 |
| Mental health | 0.069 | 0.127 | <.001 |
| Age | -0.008 | -0.127 | <.001 |
| Sex | 0.142 | 0.087 | <.001 |
| Ethnicity | -0.258 | -0.110 | <.001 |
| Geographical area | -0.009 | -0.010 | 0.525 |
| Motivations (M) |  |  |  |
| Socialising | 0.028 | 0.040 | 0.006 |
| Loneliness | 0.106 | 0.099 | <.001 |
| Living alone | -0.002 | -0.012 | 0.408 |
| SES | -0.037 | -0.040 | 0.097 |
| Physical Health | -0.200 | -0.073 | 0.001 |
| Mental health | 0.102 | 0.195 | <.001 |
| Age | -0.001 | -0.023 | 0.135 |
| Sex | 0.001 | 0.001 | 0.945 |
| Ethnicity | -0.171 | -0.076 | <.001 |
| Geographical area | -0.015 | -0.017 | 0.227 |
| Physical health |  |  |  |
| SES | 0.520 | 1.553 | <.001 |
| Age | 0.017 | 0.781 | <.001 |
| Mental health |  |  |  |
| Age | -0.019 | -0.166 | <.001 |
| Sex | 0.294 | 0.098 | <.001 |
|  |  |  |  |
| Latent variables: |  |  |  |
| Income |  |  |  |
| SES | 1 (constrained) | 0.619 |  |
| _cons | 4.088 | 3.083 | <.001 |
| Working |  |  |  |
| SES | 0.253 | 0.518 | <.001 |
| _cons | 1.042 | 2.597 | <.001 |
| Education |  |  |  |
| SES | 0.582 | 0.434 | <.001 |
| _cons | 4.297 | 3.909 | <.001 |
| C_Psych |  |  |  |
| C | 1 (constrained) | 0.739 |  |
| _cons | 1.910 | 1.885 | <.001 |
| C_Phys |  |  |  |
| C | 0.759 | 0.665 | <.001 |
| _cons | 1.745 | 2.044 | <.001 |
| O_Soc |  |  |  |
| O | 1 (constrained) | 0.705 |  |
| _cons | 1.578 | 1.403 | <.001 |
| O_Phys |  |  |  |
| O | 0.763 | 0.604 | <.001 |
| _cons | 1.933 | 1.928 | <.001 |
| M_Aut |  |  |  |
| M | 1 (constrained) | 0.806 |  |
| _cons | 2.217 | 2.353 | <.001 |
| M_Ref |  |  |  |
| M | 0.990 | 0.744 | <.001 |
| _cons | 2.030 | 2.008 | <.001 |
| Illness |  |  |  |
| Physical health | 1 (constrained) | 0.715 |  |
| _cons | -0.092 | -0.238 | <.001 |
| Pain |  |  |  |
| Physical health | 2.025 | 0.713 | <.001 |
| _cons | 0.999 | 1.280 | <.001 |
| Mobility |  |  |  |
| Physical health | 0.575 | 0.649 | <.001 |
| _cons | -0.093 | -0.382 | <.001 |
| Stress |  |  |  |
| Mental health | 1 (constrained) | 0.642 |  |
| _cons | 7.679 | 3.371 | <.001 |
| Depression |  |  |  |
| Mental health | 1.258 | 0.694 | <.001 |
| _cons | 4.646 | 1.752 | <.001 |
| Anxiety |  |  |  |
| Mental health | 3.309 | 0.913 | <.001 |
| _cons | 9.134 | 1.723 | <.001 |
|  |  |  |  |
| mean(age) | 46.650 | 3.598 | <.001 |
| mean(sex) | 0.612 | 1.257 | <.001 |
| mean(ethnicity) | 0.868 | 2.566 | <.001 |
| mean(geographical area) | 1.955 | 2.351 | <.001 |
|  |  |  |  |
| var(e.socialising) | 1.163 | 0.999 |  |
| var(e.loneliness) | 0.500 | 0.984 |  |
| var(e.income) | 1.084 | 0.617 |  |
| var(e.working) | 0.118 | 0.731 |  |
| var(e.education) | 0.980 | 0.811 |  |
| var(e.live alone) | 13.339 | 0.924 |  |
| var(e.C_Psych) | 0.467 | 0.455 |  |
| var(e.C_Phys) | 0.406 | 0.557 |  |
| var(e.O_Soc) | 0.635 | 0.502 |  |
| var(e.O_Phys) | 0.639 | 0.636 |  |
| var(e.M_Aut) | 0.311 | 0.350 |  |
| var(e.bctM_Ref) | 0.457 | 0.447 |  |
| var(e.illness) | 0.072 | 0.489 |  |
| var(e.pain) | 0.300 | 0.492 |  |
| var(e.mobility) | 0.034 | 0.578 |  |
| var(e.stress) | 3.050 | 0.588 |  |
| var(e.depression) | 3.649 | 0.519 |  |
| var(e.anxiety) | 4.684 | 0.167 |  |
| var(e.SES) | 0.602 | 0.894 |  |
| var(e.C) | 0.438 | 0.781 |  |
| var(e.O) | 0.556 | 0.885 |  |
| var(e.M) | 0.541 | 0.938 |  |
| var(e.Physical health) | 0.318 | 4.211 |  |
| var(e.Mental health) | 2.056 | 0.962 |  |
| var(age) | 168.108 | 1.000 |  |
| var(sex) | 0.237 | 1.000 |  |
| var(ethnicity) | 0.114 | 1.000 |  |
| var(geographical area) | 0.692 | 1.000 |  |
|  |  |  |  |
| cov(e.socialising,e.loneliness) | -0.144 | -0.189 | <.001 |
| cov(e.socialising,e.living alone) | -0.419 | -0.106 | <.001 |
| cov(e.loneliness,e.living alone) | -0.448 | -0.174 | <.001 |
| cov(e.SES,e.Physical health) | -0.395 | -0.903 | <.001 |
| cov(e.SES,e.Mental health) | -0.256 | -0.230 | <.001 |
| cov(e.C,e.O) | 0.459 | 0.930 | <.001 |
| cov(e.C,e.M) | 0.419 | 0.860 | <.001 |
| cov(e.O,e.M) | 0.458 | 0.836 | <.001 |
| cov(e.Physical health,e.Mental health) | 0.246 | 0.304 | <.001 |
| cov(age,sex) | -0.148 | -0.023 | <.001 |
| cov(age,ethnicity) | 0.766 | 0.175 | <.001 |
| cov(age,geographical area) | 2.319 | 0.215 | <.001 |
| cov(sex,ethnicity) | 0.000 | 0.000 | <.001 |
| cov(sex,geographical area) | 0.003 | 0.008 | <.001 |
| cov(ethnicity, geographical area) | 0.049 | 0.175 | <.001 |

*Notes: var=variance, cov=covariance*
